# Clinical utility of renal and urinary tract point-of-care ultrasound in the neonatal intensive care unit: insights from a case series

**DOI:** 10.3389/fped.2026.1880755

**Published:** 2026-09-03

**Authors:** Gayatri Morajkar, Sujata Deshpande, Sachin Verma, Vivek Goyal, Jayanta Hazarika, Prasoon Bansal, Yogen Singh, Pradeep Suryawanshi

**Affiliations:** 1 Department of Neonatology, MotherNest Hospital, Dombivali, India; 2 Department of Neonatology, Bharati Vidyapeeth Deemed University Medical College and Hospital, Pune, Maharashtra, India; 3 Department of Pediatrics, Vivekananda Polyclinic and Institute of Medical Sciences, Lucknow, India; 4 Department of Pediatrics and Neonatology, Janki Children Hospital, Hisar, Haryana, India; 5 Department of Pediatrics and Neonatology, Nagaon Advanced Medical Center, Assam, India; 6 Division of Neonatology, Department of Pediatrics, University of California, UC Davis Children's Hospital, Sacramento, Sacramento, CA, United States

**Keywords:** adrenal, bladder, neonatal intensive care unit, neonate, point-of-care ultrasonography, renal, ultrasound

## Abstract

**Background:**

Clinical decision-making in acutely ill neonates often depends on the timely identification of the underlying pathology. Bedside point-of-care ultrasound (POCUS), performed by the treating clinician, allows immediate correlation of imaging findings with evolving clinical status. However, its role in evaluating renal and urinary tract abnormalities in neonates remains relatively underexplored and is not yet widely integrated into routine practice.

**Methods:**

This multicenter case series included data from four tertiary neonatal intensive care units in India between January 2023 and March 2026. Neonates with acute clinical presentations in whom clinician-performed POCUS identified significant renal, adrenal, or urinary tract abnormalities were included. Ultrasound findings were interpreted alongside clinical and laboratory data and corroborated with formal imaging where required.

**Results:**

Seven neonates with diverse clinical presentations were evaluated. POCUS identified a spectrum of infective, obstructive, hemorrhagic, and vascular pathologies involving the kidneys, bladder, and adrenal glands. Early detection with POCUS informed timely therapeutic decisions, including initiation of targeted antimicrobial or antifungal therapy, anticoagulation, relief of urinary obstruction, and appropriate conservative management. Serial examinations enabled ongoing assessment of disease evolution and response to treatment.

**Conclusion:**

Clinician-performed renal and urinary tract POCUS provides actionable diagnostic information in acutely ill neonates and supports prompt, targeted management at the bedside. Its use may enhance clinical decision-making, particularly in time-sensitive situations where immediate access to comprehensive imaging is limited. These findings support incorporation of renal–urinary tract POCUS into structured neonatal POCUS training programs.

## 1 Introduction

Bedside ultrasound imaging is considered the “fifth pillar” of clinical examination, alongside inspection, palpation, percussion, and auscultation (1). In neonates, where decision-making is often time-sensitive, point-of-care ultrasound (POCUS) reduces diagnostic uncertainty, provides real-time longitudinal assessment, and supports early diagnosis and management (2).

The clinical presentation of urological conditions in newborns is often subtle, with non-localizing features, leading to challenges in diagnosis. Delayed recognition may result in hemodynamic instability, acute kidney injury, and long-term renal sequelae. Ultrasonography is the first-line imaging modality for evaluating structural and functional abnormalities of the renal and urinary tract system (3). However, access to formal radiology services may be limited in acute scenarios, particularly in resource-limited centers and situations. In such circumstances, clinician-performed bedside ultrasound can provide rapid and focused clinical information to guide immediate decision-making (4).

Recently, the European Society of Paediatric and Neonatal Intensive Care (ESPNIC) published evidence-based guidelines for the implementation of POCUS across various organ systems in neonatal intensive care units (NICUs) (5). These guidelines describe selected renal ultrasound applications, such as hydronephrosis and obstructive uropathy. However, the broader application of bedside renal and urinary tract imaging is still underexplored.

In this context, our case series highlights the diagnostic utility and clinical implications of renal and urinary tract POCUS in the management of sick neonates in the NICU.

## 2 Materials and methods

This multicenter case series included data from four tertiary care NICUs in India between January 2023 and March 2026. Cases were identified retrospectively by reviewing archived ultrasound images stored during this period. Symptomatic neonates in whom bedside imaging of the renal, suprarenal, or urinary tract by the treating neonatal physicians revealed significant ultrasound findings were included. Neonates with antenatally detected structural anomalies of the urinary tract were excluded. Bedside ultrasound examinations were performed by clinicians who had a minimum of 6 months of structured training in neonatal ultrasound, using the ultrasound machine and the transducers available at the participating NICUs. High-frequency linear transducers (ranging from 4 to 18 MHz) were primarily used for imaging the kidneys, urinary bladder, and adrenals. Other probes used included curved (4 to 10 MHz) and hockey-stick probes (for femoral vessel). Color imaging and Doppler imaging were performed when vascular assessment was clinically indicated. Examinations were performed at the bedside with the infant in an incubator or radiant warmer, predominantly in the supine position. Gentle probe angulation and minimal repositioning (lateral decubitus or prone positioning) were used to optimize visualization while minimizing handling. Findings were corroborated by formal radiological assessment when indicated. Clinical data, laboratory results, and radiological reports were retrieved from electronic and paper medical records. Patient identifiers were kept confidential. Written informed consent was obtained from the parents/legal guardians of the cases included for the publication of any potentially identifiable data or images included in this manuscript.

## 3 Results

We identified seven neonates admitted to the NICU who underwent POCUS by the treating physician, which revealed significant radiological findings in the kidneys, bladder, or adrenals. The conditions identified included pyonephrosis, renal fungal balls, transient urinary retention, bladder hematoma, adrenal hemorrhage, and renovascular thrombosis. In each case, clinician-performed bedside ultrasound directly influenced clinical decision-making and subsequent management. The clinical and radiological characteristics of these cases are summarized in Table 1.

**Table 1:** Summary of clinical course and radiological findings of study neonates.

Case	Clinical presentation	Bedside POCUS findings	Clinical management after POCUS and time to radiologic confirmation	Final diagnosis
1	Sepsis, deranged renal function tests, abnormal urinalysis	Bilateral dilatation of collecting system with internal echogenic debris within the renal pelvis	Continued empirical antibiotics → radiologic confirmation: ∼12 h after bedside POCUS → serial renal ultrasound surveillance	Bilateral pyonephrosis
2	Sepsis and acute renal failure	Echogenic lesions with linear foci in the renal pelvis	Antifungal therapy initiated → formal radiologic confirmation: ∼12 h after bedside POCUS → serial renal ultrasound surveillance	Renal fungal balls
3	Non-passage of urine for 6 h in a postoperative newborn	Well-distended urinary bladder containing urine (∼20 mL)	Urinary catheterization performed with immediate drainage; no formal imaging required	Transient urinary retention
4	Gross hematuria and drop in hemoglobin, postsuprapubic aspiration	Intraluminal echogenic mass in the bladder with heterogeneous areas within	Packed red blood cell transfusion and bladder irrigation with normal saline and hemocoagulase → radiologic confirmation: ∼1 h after bedside POCUS → follow-up imaging	Bladder hematoma
5	Perinatal asphyxia, acute drop in hemoglobin	Bilateral suprarenal masses with hypoechoic areas within	Conservative management → radiologic confirmation:∼6 h after bedside POCUS → serial hemoglobin and ultrasound	Bilateral adrenal hemorrhage
6	Perinatal asphyxia, progressive decline in hemoglobin over a week	Large bilateral suprarenal masses with internal hypoechoic areas	Conservative management → radiologic confirmation: ∼12 h after bedside POCUS → serial ultrasound follow-up	Bilateral adrenal hemorrhage
7	Congenital diaphragmatic hernia, umbilical arterial catheterization, limb ischemia	Hypoechoic structure within the left femoral artery with residual flow; left renal artery Doppler—reduced peak systolic velocity and reversed end-diastolic flow	Unfractionated heparin therapy initiated → radiologic confirmation: ∼5 h after bedside POCUS → CT angiography at 48 h	Arterial thrombus with renal infarction

### 3.1 Case 1

A late-preterm female neonate (36 + 1 weeks, 2,700 g at birth) presented on day 15 of life with seizures, altered sensorium, hypoglycemia, and shock. She received non-invasive respiratory support, intravenous (IV) fluids, dextrose, anticonvulsants, and empirical antibiotics for suspected sepsis. Investigations showed severe thrombocytopenia (20 × 10^9^/L), elevated C-reactive protein (CRP 174 mg/L), renal dysfunction (serum urea 31.4 mmol/L, creatinine 159 μmol/L), and numerous pus cells on urine microscopy. Cerebrospinal fluid (CSF) revealed pleocytosis (36 cells/ HPF; 90% neutrophils), suggestive of bacterial meningitis.

**Figure 1:**
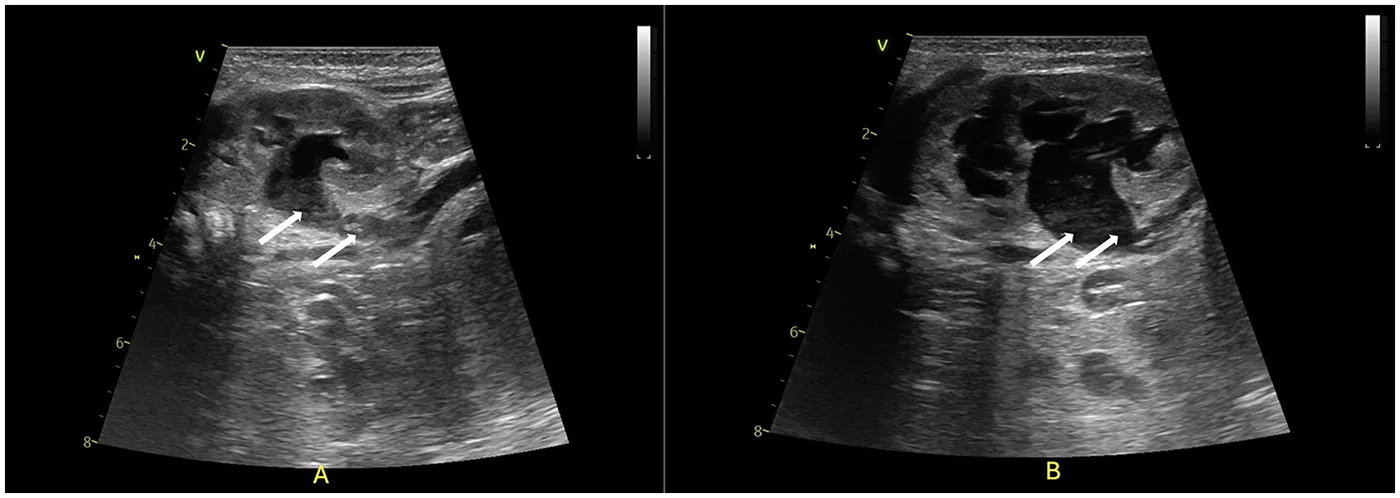
**(A)** Longitudinal view of the right kidney demonstrating dilatation of the pelvicalyceal system and ureter with echogenic debris within the renal pelvis (arrows). **(B)** Transverse view of the right kidney showing marked dilatation of the pelvicalyceal system, with echogenic debris within the renal pelvis (arrow), noted to be mobile on real-time imaging.

After stabilization, bedside POCUS was performed approximately 3–4 h after the admission. Cranial ultrasound showed a large left frontoparietal intraparenchymal hemorrhage with midline shift. In view of abnormal renal parameters and non-availability of a radiologist at the time, a bedside renal scan was performed by the neonatal physician. This revealed dilatation of the bilateral collecting systems with echogenic debris in the renal pelvis, suggestive of hydroureteronephrosis with pyonephrosis (Figures 1A,B). Formal radiological evaluation subsequently demonstrated bilateral grade 1 hydronephrosis and possible pyonephrosis, with bilateral kidneys of normal size and echogenicity. Empirical broad-spectrum antimicrobial therapy, initiated for suspected late-onset sepsis, was continued. Bedside renal POCUS provided early evidence of pyonephrosis, establishing the presence of a suppurative upper urinary tract infection that could not be identified by laboratory investigations alone. The diagnosis also prompted serial renal ultrasound surveillance to monitor treatment response and assess the need for urinary drainage should obstruction progress. Urine and CSF cultures grew *Escherichia coli*; the infant received culture-sensitive antibiotics for 21 days. Serial scans showed progressive resolution without long-term renal sequelae.

### 3.2 Case 2

A late-preterm male infant (34 weeks, 1,610 g at birth), with a history of NICU stay for 1 month and successful discharge home, was readmitted at 2 months of life, with a history of vomiting, loose stools, and decreased urine output. He had abdominal distension, oliguria, and a positive sepsis screen with elevated CRP. Despite IV fluids, antibiotics, and supportive treatment, the infant developed anuria and worsening azotemia (serum creatinine 610 µmol/L) over the following 12–24 h.

**Figure 2:**
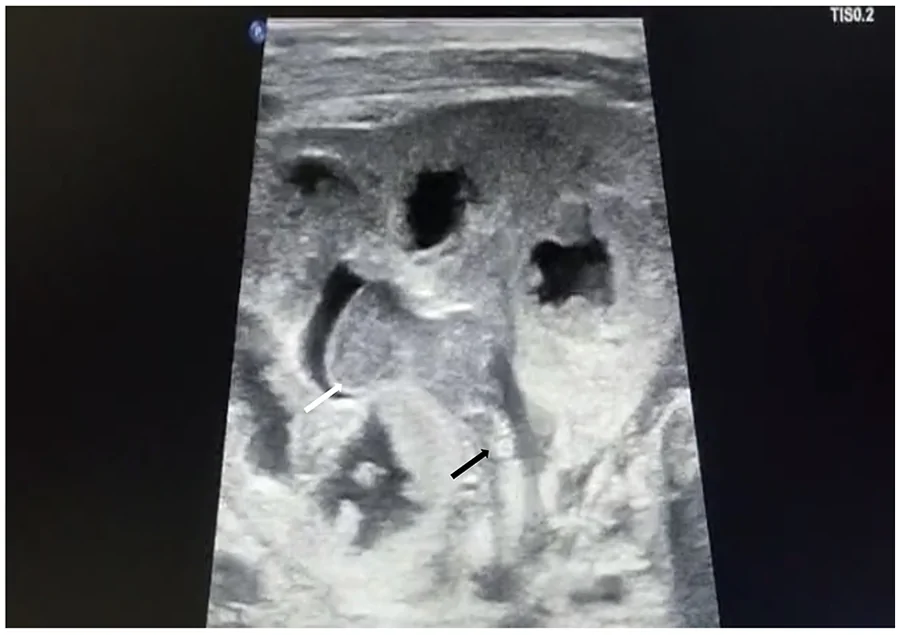
Transverse view of the kidney showing hyperechoic renal parenchyma, with echogenic material within the renal pelvis (white arrow), along with linear echogenic foci (black arrow).

This acute deterioration prompted bedside renal POCUS by the treating physician, which revealed hyperechoic renal parenchyma and dense echogenic obstructive lesions with linear foci in the renal pelvis, suggestive of fungal balls (Figure 2). Empirical antifungal therapy was initiated. Radiological evaluation the subsequent day revealed bilateral bulky kidneys, increased parenchymal echogenicity with loss of corticomedullary differentiation, mild to moderate bilateral hydronephrosis, linear echogenic foci in the collecting system in the bilateral kidneys, and sediments, suggestive of pyonephrosis and fungal infection. A micturating cystourethrogram excluded bladder outlet obstruction, while MRI urography showed bilateral renal enlargement with dilated calyces, suggestive of pyonephrosis without hydroureter.

Peritoneal dialysis was required for 48 h until urine output improved and creatinine normalized. Blood culture grew *Candida albicans*. Cranial and cardiac POCUS screening, as well as fundus examination, showed no evidence of fungal dissemination. Serial imaging showed lesion regression with clinical improvement. Antifungal therapy was continued for 6 weeks and stopped after sterile cultures.

### 3.3 Case 3

A term female neonate (38 weeks, 3,000 g at birth) was diagnosed with left-sided congenital lobar emphysema in the early neonatal period. She underwent a left posterolateral thoracotomy with left upper lobectomy on day 37 of life for persistent respiratory distress requiring respiratory support. The procedure was uneventful, and the neonate was transferred to the NICU for postoperative care.

**Figure 3:**
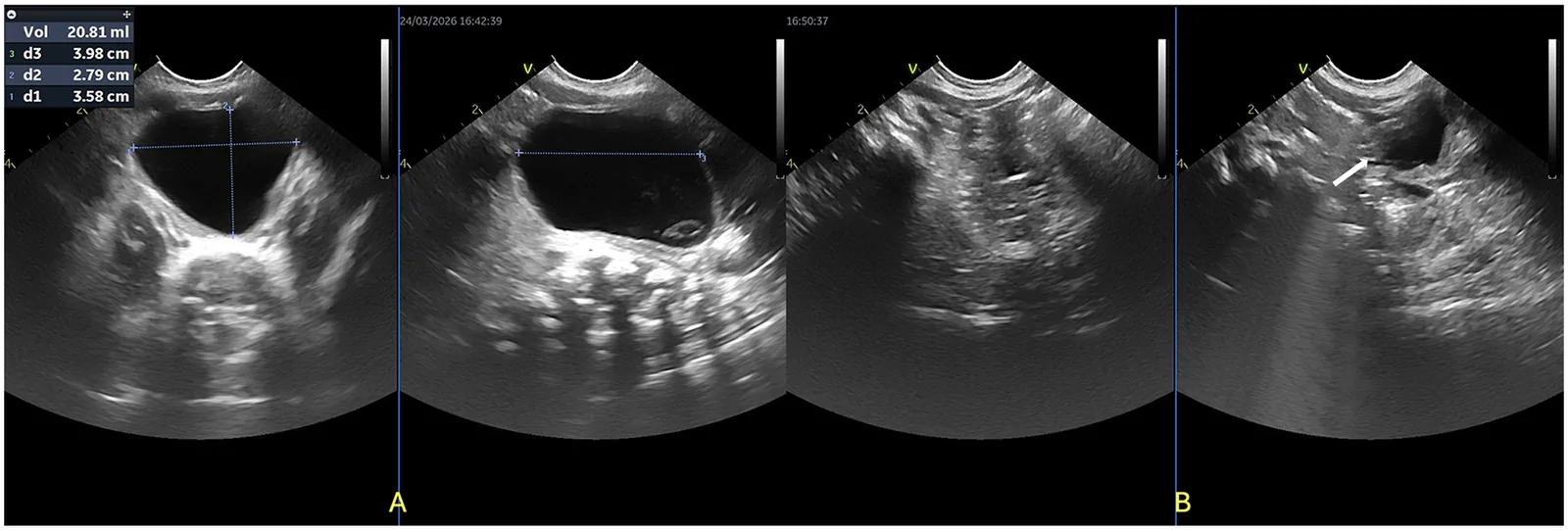
**(A)** Bladder POCUS (transverse and longitudinal views) demonstrating a well-filled urinary bladder, with an estimated volume of approximately 20 mL. **(B)** Bladder ultrasound demonstrating a collapsed urinary bladder following catheterization, with the urinary catheter *in situ* (arrow).

Approximately 6 h postoperatively, she was noticed to have absent urine output. On clinical examination, a palpable bladder was suspected, although the finding was uncertain. Bedside bladder POCUS performed by the neonatal provider demonstrated a well-distended urinary bladder with an estimated urine volume of approximately 20 mL (Figure 3A). Urinary catheterization was performed, yielding around 25 mL of urine. Postvoid POCUS confirmed complete emptying of the bladder (Figure 3B). Over the next 24 h, the infant passed adequate volumes of urine and continued to void well after catheter removal. Serum creatinine and electrolytes were within normal limits. As the bedside findings were immediately confirmed by successful bladder catheterization and complete symptom resolution, formal radiological assessment was not considered.

### 3.4 Case 4

A preterm male infant (30 weeks, 1,150 g at birth) was born via vaginal delivery to a 28-year-old G3P2 mother following spontaneous preterm labor. In the NICU, the infant was managed with non-invasive respiratory support, parenteral nutrition, nasogastric feeding, and empiric antibiotics, which were discontinued following sterile culture results.

**Figure 4:**
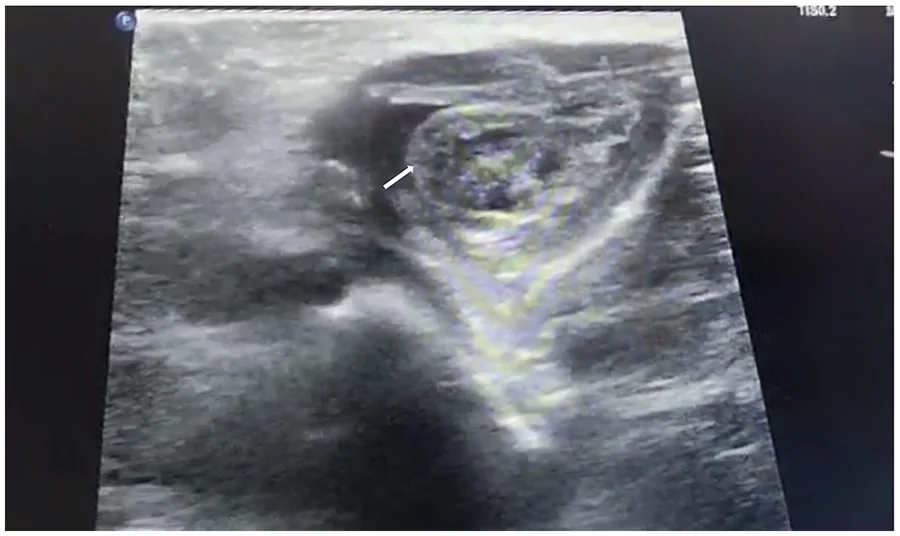
**(A)** Ultrasound image of the urinary bladder demonstrating an intraluminal, well-defined heterogeneous mass (arrow), with surrounding urine.

On day 8 of life, the neonate developed feed intolerance and recurrent apnea, necessitating evaluation for late-onset sepsis. Suprapubic urinary aspiration (SPA) was performed to rule out urinary tract infection (UTI). Within 12 h postprocedure, the infant developed gross hematuria with an acute drop in hemoglobin (126 to 96 g/L). Platelet count and coagulation profile were within normal limits. Bedside bladder POCUS showed an intraluminal echogenic mass with heterogeneous areas within, suggestive of intravesical hematoma (Figure 4). The kidneys and the rest of the urinary tract appeared normal. Findings were confirmed by a radiologist. The neonate was managed with packed red cell transfusion and bladder irrigation using normal saline and hemocoagulase. Hematuria resolved without recurrence, and follow-up imaging at 3 weeks showed complete resolution.

### 3.5 Cases 5 and 6

Two newborns with perinatal asphyxia and an unexplained fall in hemoglobin were evaluated for the underlying cause.

**Figure 5:**
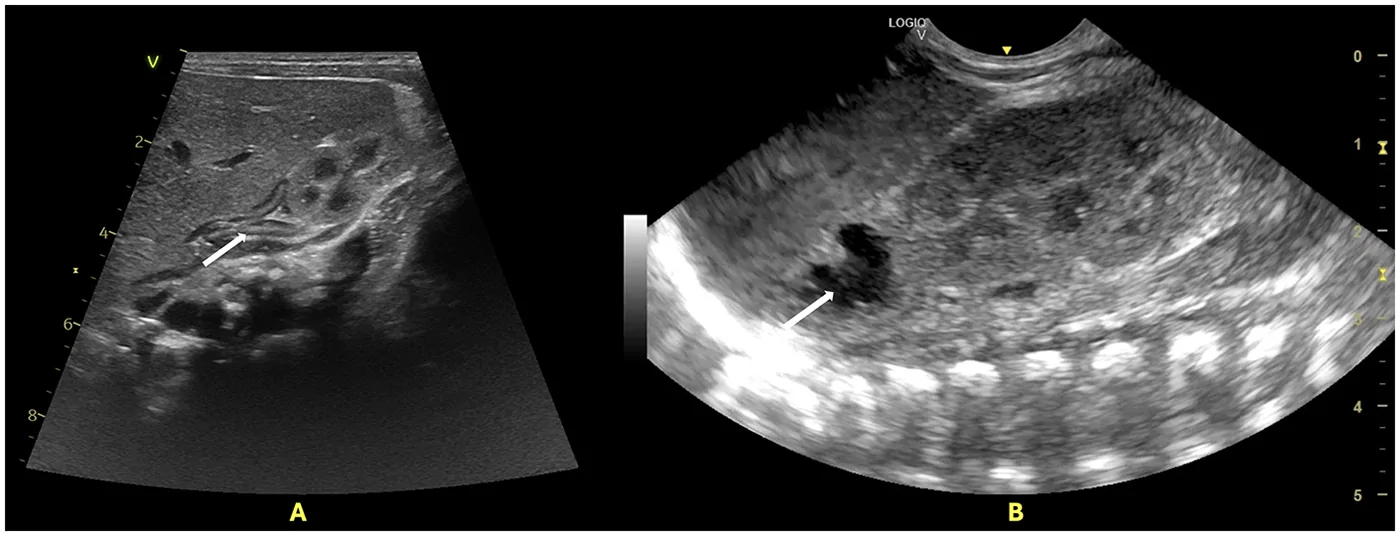
**(A)** Normal neonatal adrenal gland demonstrating a typical Y-shaped hypoechoic structure (arrow) with an echogenic central medulla at the upper pole of the kidney. **(B)** Longitudinal view of the right suprarenal region showing a suprarenal mass with internal hypoechoic areas (arrow).

The first case was a preterm male neonate (32 + 3 weeks, 1,430 g at birth), delivered by emergency cesarean section for decreased fetal movements and Doppler abnormalities. The neonate was non-vigorous at birth and required positive-pressure ventilation. Cord blood gas revealed severe metabolic acidosis. A significant drop in hemoglobin (from 177 to 121 g/L) was noted within the first 24 h of life. In the absence of hemolysis and with a normal platelet count and coagulation profile, screening POCUS was performed to evaluate for internal hemorrhage. Cranial ultrasound showed no evidence of intracranial bleed. Abdomen and renal POCUS revealed bilateral suprarenal masses with hypoechoic areas within, suggestive of adrenal hemorrhage (Figure 5B). A normal sonographic appearance of the neonatal adrenal gland is shown alongside (Figure 5A) for comparison. Formal radiologic assessment confirmed bilateral adrenal hemorrhage, demonstrating well-defined heterogeneous, predominantly hypoechoic bilateral suprarenal lesions replacing the adrenal gland, with internal low-level echoes and absence of internal vascularity on color Doppler. Both kidneys appeared normal. Follow-up bedside ultrasonography performed 2 weeks later demonstrated reduction in both adrenal lesions, consistent with resolving hemorrhage.

**Figure 6:**
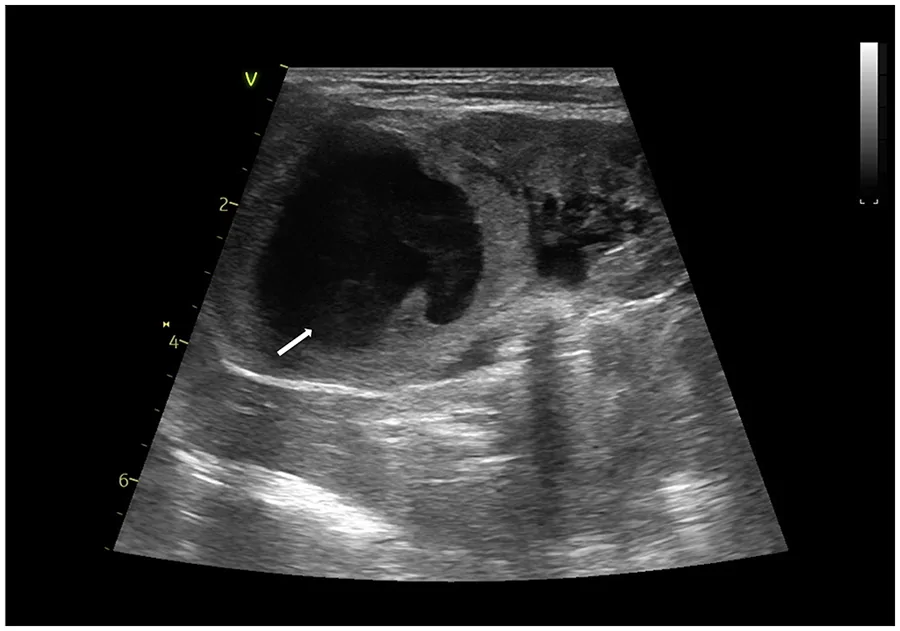
Ultrasound image demonstrating a large right-sided suprarenal mass with internal hypoechoic areas (arrow).

The second case was a term male neonate (38 weeks, 3,300 g at birth), admitted to the NICU with perinatal asphyxia. Admission blood gas revealed severe metabolic acidosis. During the NICU stay, the neonate developed seizures and was managed with mechanical ventilation, anticonvulsants, and therapeutic hypothermia. Cranial imaging suggested severe hypoxic–ischemic injury. Despite clinical stabilization, hemoglobin declined from 200 to 100 g/L by day 9, without evidence of hemolysis or external blood loss. Repeat cranial POCUS did not reveal any hemorrhage, but screening ultrasound of the abdomen and kidneys revealed large bilateral suprarenal masses, consistent with adrenal hemorrhage (Figure 6). Formal radiologist-performed ultrasonography confirmed bilateral adrenal hemorrhage, demonstrating well-defined hypoechoic lesions with internal low-level echoes.

Both infants were managed conservatively with monitoring and remained stable, without evidence of adrenal insufficiency.

### 3.6 Case 7

A preterm male twin (34 weeks, 2,100 g at birth) with antenatally diagnosed left-sided congenital diaphragmatic hernia was managed with high-frequency ventilation, inotropes, and inhaled nitric oxide for persistent pulmonary hypertension of the newborn. An umbilical arterial catheter (UAC) was placed for invasive arterial blood pressure monitoring. He underwent surgical repair on day 5.

**Figure 7:**
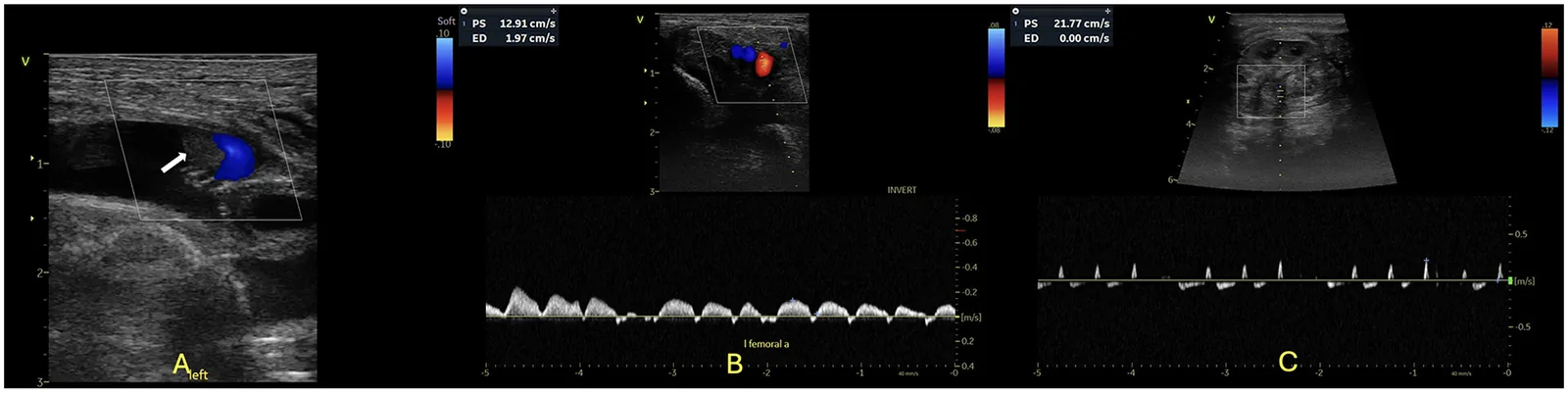
**(A)** Intravascular hypoechoic mass with residual flow within the left femoral artery, suggestive of a thrombus. **(B)** Spectral Doppler of the left femoral artery demonstrating markedly reduced peak systolic velocity. **(C)** Left renal artery Doppler with reduced peak systolic velocity and reversal of end-diastolic flow. (**A,C**) Reprinted with permission from Abdominal Aortic Thrombosis in a Preterm Baby With Congenital Diaphragmatic Hernia. Cureus 17(8): e89401. doi: 10.7759/cureus.89401 by: Goyal V, Jain N, Goyal M (August 05, 2025), licensed under under the Creative Commons Attribution License (CC BY 4.0).

On day 11, the neonate developed left lower-limb discoloration with absent peripheral pulses, suggestive of arterial ischemia. Despite UAC removal, limb perfusion did not improve. Bedside ultrasound performed by the treating physician showed a mobile intraluminal hypoechoic structure with residual flow within the left femoral artery (Figure 7A) and markedly reduced peak systolic velocity on spectral Doppler (Figure 7B), suggestive of a partially occluding thrombus in the left femoral artery. Renal ultrasound demonstrated reduced blood flow in the left kidney. Left renal artery Doppler was consistent with renal infarction, with reduced peak systolic velocity and reversed end-diastolic flow (Figure 7C), raising suspicion of the thrombus extending into renal branches of the abdominal aorta. There was no systemic hypertension, and renal function tests remained within normal limits. These findings were subsequently confirmed by formal radiological evaluation, which revealed mild narrowing of right renal artery with normal right renal perfusion, completely thrombosed left renal artery with no intrarenal flow to left kidney, and partial thrombosis of the infrarenal abdominal aorta. Anticoagulant therapy with unfractionated heparin was initiated, with improvement in limb perfusion over 48–72 h. Computed tomography (CT) angiography later confirmed left renal artery thrombosis with global infarction of the left kidney, partial thrombosis of the infrarenal abdominal aorta, and iliac artery narrowing.

The infant was discharged on day 29 of life on subcutaneous enoxaparin with anti-Xa factor monitoring. Follow-up Doppler study at 2 months demonstrated persistent absence of flow in the left renal artery with reduced renal size.

## 4 Discussion

Neonatal renal and urinary tract pathologies often present with non-specific features such as oliguria, sepsis, or unexplained anemia, making diagnosis in critically ill newborns challenging. In acute settings, particularly where round-the-clock radiology services are unavailable, delays in imaging may hinder targeted therapy. Our case series highlights the role of clinician-performed renal and urinary tract POCUS in symptomatic neonates, providing rapid bedside diagnostic clues and guiding timely management.

In cases of pyonephrosis and renal fungal balls, bedside imaging enabled early identification of urinary tract infection, guided antimicrobial treatment, and informed follow-up planning. Bladder POCUS identified urinary retention in a postoperative infant with transient anuria and detected an intravesical hematoma in another neonate with hematuria, facilitating timely management and resolution. In neonates with unexplained anemia, POCUS revealed bilateral adrenal hemorrhage, supporting conservative management. In the case of arterial thrombosis, bedside Doppler ultrasound identified a thrombus with renal infarction, enabling anticoagulation and prevention of lower-limb ischemia.

Bedside renal ultrasound is performed in supine, lateral, or prone positions using a linear/curvilinear high-frequency (≥9 MHz) probe (6). Each kidney is assessed in longitudinal and transverse planes for presence, location, size, echogenicity, corticomedullary differentiation, collecting system abnormalities, and perfusion. In neonates, the renal cortex is normally more echogenic than the liver and spleen, with relatively hypoechoic medulla (7). Adrenal glands, assessed in lateral or prone positions, appear as hypoechoic L-, V-, or Y-shaped structures capping the upper renal pole (Figure 5A) (8). The bladder appears as an anechoic, fluid-filled pelvic structure, with imaging performed in the supine position via a suprapubic approach in sagittal and transverse planes (9). Assessment includes contents, wall thickness, and volume estimation (length × width × height × 0.53) (7, 10). Renal artery Doppler evaluates vascular patency and perfusion, with resistive index values >0.70 considered abnormal (11).

### 4.1 Neonatal pyonephrosis

Pyonephrosis is an infected form of hydronephrosis characterized by suppurative destruction of the renal parenchyma, potentially leading to loss of renal function. While older children typically present with fever, chills, and flank pain, neonates often have non-specific features ranging from asymptomatic bacteriuria to severe sepsis or septic shock (12–14). It is considered a urological emergency and may require urgent drainage by percutaneous nephrostomy (14).

Ultrasound plays a crucial role in the diagnosis of pyonephrosis, with echogenic debris being the most reliable finding (sensitivity 90%, specificity 97%) (15). Dilatation of the pelvicalyceal system may be observed secondary to underlying obstruction. In a previously reported case of neonatal pyonephrosis presenting with septic shock, ultrasonography guided diagnosis and management with percutaneous nephrostomy and antibiotics (13). In our case, bedside imaging identified echogenic debris in the pelvis with hydroureteronephrosis, establishing the presence of a suppurative upper urinary tract infection. Early recognition of pyonephrosis remains clinically important as it represents a potentially drainable complication requiring close imaging surveillance to monitor treatment response and determine the need for urinary drainage if obstruction progresses. As the renal parenchyma was preserved and serial imaging revealed progressive resolution, the infant was managed conservatively with antimicrobial therapy.

### 4.2 Renal fungal balls

Fungal balls are uncommon but serious complications of fungal UTIs in newborns. Clinical manifestations range from asymptomatic funguria to severe sepsis and multiorgan dysfunction, most commonly due to *Candida* spp. (16). Predisposing factors include prematurity, prolonged use of invasive interventions such as indwelling urinary catheters, central lines, parenteral nutrition, invasive ventilation, and extensive antibiotic use (16, 17). Fungal balls are aggregates of necrotic debris mixed with proliferating fungal elements within the renal parenchyma or collecting system, potentially causing urinary tract obstruction, persistent fungemia despite antifungal therapy, and increased morbidity and mortality (16).

Ultrasound findings suggestive of renal involvement include renomegaly, diffuse parenchymal echogenicity, focal linear or wedge-shaped densities, multiple non-shadowing echodense rounded opacities within the renal calyces or collecting system, and hydronephrosis due to obstruction. In the setting of systemic candidemia, these features are suggestive of renal candidiasis (17, 18). In our case, bedside renal POCUS detected fungal balls in the pelvis, enabling early presumptive antifungal therapy prior to culture confirmation, with subsequent resolution on serial scans. A similar report by Stocker et al. described bilateral renal non-obstructive fungal balls identified by ultrasound and successfully managed with antifungal therapy (19).

### 4.3 Transient urinary retention

Evaluation of anuria or urinary retention is a key application of renal and bladder ultrasound in neonates (5, 20). This can be performed reliably by clinicians with minimal training (21). Visualization of a distended bladder may suggest urinary retention, whereas an empty bladder supports true oliguria, thereby aiding diagnosis and fluid management in states of low urine output (22). Bladder POCUS also helps confirm adequate bladder filling prior to catheterization, reducing unnecessary or failed attempts (21, 23, 24).

Pai et al. described bedside bladder ultrasound in two neonates with oliguria: One encephalopathic infant on paralytics had a distended bladder and was managed with catheterization, while a postoperative newborn with a low-volume bladder was treated with fluid boluses (25). Similarly, in our case, bedside bladder POCUS in a postoperative neonate showed a fluid-filled bladder, suggestive of transient urinary retention, likely related to perioperative medications. Thus, bladder ultrasound, with its relatively short learning curve, is a valuable tool in the prompt assessment of neonates with reduced urine output.

### 4.4 Bladder hematoma

Suprapubic aspiration is a sterile method to obtain an uncontaminated urine sample in suspected UTI and is considered the gold standard, as it bypasses the normally colonized distal urethra (26). It involves insertion of a needle through the lower abdominal wall into the bladder (26, 27). Although the procedure is generally safe, reported complications include hematuria, bladder wall or supravesical hematoma, bowel perforation, and anterior abdominal wall abscess (28–31).

Ultrasound allows rapid bedside evaluation in infants who develop gross hematuria after SPA. It can identify bladder wall injury, intraluminal clots, and potential urinary obstruction (29, 32). In our case, bedside ultrasound revealed an intravesical hematoma, enabling conservative management with close monitoring. On ultrasound, such hematomas appear heterogeneous with mixed echogenicity (32). A similar complication was reported by Morrell et al., where a bladder wall hematoma, along with urinary tract obstruction, was detected by intravenous pyelography (28). In contrast, serial ultrasound in our case demonstrated progressive resolution of the hematoma, highlighting the advantage of POCUS as a safe, radiation-free, repeatable bedside tool in neonates.

### 4.5 Adrenal hemorrhage

The true prevalence of neonatal adrenal hemorrhage (NAH) is likely underreported, as many cases are asymptomatic or undiagnosed. With the increasing use of ultrasonography, detection rates of NAH have increased. The adrenal gland in newborns is vulnerable because of its larger size at birth and high vascularity (33). Risk factors include birth asphyxia, traumatic delivery, sepsis, coagulopathy, and macrosomia (34). Clinical manifestations vary and may include prolonged jaundice, abdominal mass, anemia, scrotal swelling, or hypertension (33).

In our cases, perinatal asphyxia and an unexplained fall in hemoglobin prompted bedside imaging of the adrenals. In the early phase, ultrasonography identifies NAH as a large heterogeneous suprarenal mass, with variable internal echoes. Serial scans demonstrate size reduction with central hypoechogenicity due to clot lysis, producing a pseudocystic appearance; calcifications may appear after 1–2 weeks (33, 34). The absence of internal vascularity on color Doppler, together with gradual regression on serial ultrasonography, helps differentiate NAH from neuroblastoma, an important differential diagnosis of adrenal mass in the neonatal period (35, 36). In our cases, the diagnosis of adrenal hemorrhage was supported by the clinical setting of perinatal asphyxia presenting with drop in hemoglobin, absence of internal vascularity on Doppler imaging, and progressive regression on serial scans. Gyurkovits et al. reported 74 neonates with NAH on routine scans at 48 h of life in healthy newborns. Only one infant developed adrenal insufficiency, while the remaining infants were managed conservatively (37). The clinical course of our cases was comparable, supporting conservative management in hemodynamically stable infants without adrenal insufficiency.

### 4.6 Renal artery thrombosis

Renal artery thrombosis is a rare but serious complication in newborns. Risk factors include indwelling UAC, maternal diabetes, sepsis, dehydration, coagulopathy, patent ductus arteriosus, shock, and cannulation of the femoral artery (6, 14). The affected kidney often progresses to global infarction and atrophy. Clinical presentation ranges from asymptomatic to hypertension, hematuria, oliguria, and limb ischemia (14, 38, 39).

Ultrasound is central to the initial evaluation of suspected renovascular thrombosis. Grayscale findings may be subtle; in the initial phase, the kidney may appear normal in size and hyperechoic due to edema and hemorrhage, with loss of corticomedullary differentiation as ischemia progresses. A fresh thrombus may appear hypoechoic or isoechoic and may sometimes be missed on grayscale. Color Doppler is essential to demonstrate vascular patency and identify absent or reduced flow distal to the thrombus (6, 14).

Renovascular thrombosis is a recognized complication of umbilical arterial catheterization (38, 39). In our case, the presence of a UAC with cardiorespiratory instability likely contributed to thrombus formation in the femoral and renal arteries. Identification of the thrombus by ultrasound and initiation of anticoagulation therapy likely preserved limb circulation. A comparable case by Kavaler and Hensle described a term infant with complex congenital heart disease who presented with renal failure and shock. Doppler ultrasonography revealed a poorly perfused right kidney, later confirmed as renal artery thrombosis on diethylenetriamine pentaacetic acid scan (38).

While angiography remains the gold standard for diagnosing vascular pathology, Doppler ultrasound serves as an immediate, non-invasive tool that guides further evaluation and management.

## 5 Limitations of renal POCUS

While POCUS is a valuable bedside tool for neonatologists in the NICU, several limitations should be acknowledged. It is operator-dependent, with a steep learning curve for accurate interpretation of neonatal-specific renal pathologies, thereby requiring structured training and quality assurance (40). The neonatal kidney presents unique anatomical challenges. Prominent, hypoechoic renal pyramids can be misinterpreted as dilated calyces or cystic renal diseases, while the relatively hyperechoic cortex may mimic cortical scarring (41). Early scans (within the first 48–72 h of life) may underestimate the severity of hydronephrosis due to relative dehydration and low glomerular filtration rate (42). In addition, non-dilated ureters are often not visualized by ultrasound. Renal ultrasound also demonstrates poor sensitivity and specificity in identifying low-grade vesicoureteral reflux, renal cortical scarring, and early uncomplicated pyelonephritis (23, 42–45). Formal imaging by a radiologist remains essential to confirm findings, exclude structural abnormalities, and complement bedside POCUS assessment.

## 6 Conclusion

This case series highlights the utility of clinician-performed bedside POCUS in the early detection of renal, suprarenal, and lower urinary tract abnormalities presenting acutely in neonates. In each case, POCUS narrowed diagnostic uncertainty, improved clinical decision-making, enabled timely therapeutic decisions, and facilitated dynamic, non-invasive monitoring while supporting early referral to specialists. Comprehensive radiological imaging remains indispensable for detailed anatomical assessment and definitive diagnosis. However, in acute settings, POCUS can provide early diagnostic insights and meaningfully guide the management of sick neonates in intensive care. These advantages underscore the importance of incorporating renal–suprarenal–bladder applications into structured neonatal POCUS training frameworks.

## Data Availability

The raw data supporting the conclusions of this article will be made available by the authors, without undue reservation.
